# Predicting survival and longevity of sows using purebred and crossbred data^1^

**DOI:** 10.1093/tas/txaa073

**Published:** 2020-06-01

**Authors:** Maja W Iversen, Øyvind Nordbø, Eli Gjerlaug-Enger, Eli Grindflek, Theodorus H E Meuwissen

**Affiliations:** 1 Norsvin R&D, Hamar, Norway; 2 Norwegian University of Life Sciences, Department of Animal and agricultural sciences, Ås, Norway; 3 GENO SA, Hamar, Norway

**Keywords:** crossbreds, longevity, pigs, survival

## Abstract

Survival and longevity are very important traits in pig breeding. From an economic standpoint, it is favorable to keep the sows for another parity instead of replacing them and, from the animal’s perspective, better welfare is achieved if they do not experience health problems. It is challenging to record longevity in purebred (PB) nucleus herds because animals are more likely to be replaced based on breeding value and high replacement rates rather than inability to produce. Crossbred (CB) sows are, however, submitted to lower replacement rates and are more likely to be kept in the farm longer if they can produce large and robust litters. Therefore, the objective of this study was to investigate whether the use of CB phenotypes could improve prediction accuracy of longevity for PBs. In addition, a new definition of survival was investigated. The analyzed data included phenotypes from two PB dam lines and their F1 cross. Three traits were evaluated: 1) whether or not the sow got inseminated for a second litter within 85 d of first farrowing (Longevity 1–2), 2) how many litters the sow can produce within 570 d of first farrowing [Longevity 1–5 (LGY15)], and 3) a repeatability trait that indicates whether or not the sow survived until the next parity (Survival). Traits were evaluated both as the same across breeds and as different between breeds. Results indicated that longevity is not the same trait in PB and CB animals (low genetic correlation). In addition, there were differences between the two PB lines in terms of which trait definition gave the greatest prediction accuracy. The repeatability trait (Survival) gave the greatest prediction accuracy for breed B, but LGY15 gave the greatest prediction accuracy for breed A. Prediction accuracy for CBs was generally poor. The Survival trait is recorded earlier in life than LGY15 and seemed to give a greater prediction accuracy for young animals than LGY15 (until own phenotype was available). Thus, for selection of young animals for breeding, Survival would be the preferred trait definition. In addition, results indicated that lots of data were needed to get accurate estimates of breeding values and that, if CB performance is the breeding goal, CB phenotypes should be used in the genetic evaluation.

## INTRODUCTION

Survival and longevity of the sow are very important traits in pig breeding because replacing a sow is more expensive than keeping a sow in the herd for an extra parity (Hoge and Bates, 2011). In addition, if there is a high replacement of sows (in the absence of self-recruitment), there is a higher risk of introducing diseases into the herd, which is a welfare issue (Serenius and Stalder, 2006). To improve longevity, it is necessary to breed for sows that are able to produce many litters of healthy and robust piglets without compromising the production or health of the animal. However, data collection on longevity is currently performed only in purebred (PB) animals. This is a challenge when the goal is to improve genetic progress in crossbreds (CBs) because the trait may not be the same in PB and CB due to different effects of the genotypes, environmental differences, and potential differences in how the trait is measured (Wientjes and Calus, 2017). In addition, the recording of longevity in nucleus animals is compromised because these animals are mainly replaced based on their breeding values and high replacement rates and not due to their inability to produce. Several studies have investigated longevity traits, where the trait has either been analyzed with survival analysis models (Ducrocq and Sölkner, 1998; Vollema and Groen, 1998; Vukasinovic, 1999; Heagerty and Zheng, 2005; Engblom et al., 2009), linear models (Aasmundstad et al., 2014; Aasmundstad et al., 2015; Abell et al., 2016), or random regression models (Veerkamp et al., 1999). Another approach is to model survival using a repeatability model, where survival to the next parity is indicated as a binary trait (Meuwissen et al., 2002). Survival analysis models are useful because they are able to account for censored animals (animals that are still alive and thus do not have a final lifetime phenotype), whereas linear models do not (Vollema and Groen, 1998). Random regression models also seem robust to censoring (Veerkamp et al., 1999). On the other hand, survival analysis models are nonlinear, complex, and computationally demanding when applied to large practical data sets. Survival analysis models also require analysis using specialized software (e.g., “Survival Kit”; Ducrocq and Sölkner, 1998), which hampers multitrait evaluations together with other traits. Multitrait evaluations is a straightforward extension for linear models, which is common in routine breeding value estimations (Guo et al., 2001; Serenius and Stalder, 2006). Linear models of longevity are regularly fitted in multitrait evaluations, but heritability is often relatively low (Vollema and Groen, 1998; Aasmundstad et al., 2014).

A particular challenge with the longevity trait is that the observed longevity/culling is a product of many factors that contribute to a sow’s survival, typically functional traits, such as health and reproduction, but also production traits (Yazdi et al., 2000). Factors that have been found to have a significant effect on culling are litter size (i.e., production), herd year, age at first farrowing, and first-parity performance (Yazdi et al., 2000; Guo et al., 2001; Hoge and Bates, 2011). These factors should, therefore, be considered in the prediction model.

The goal of this study was to compare the currently used traits for longevity to a new trait, Survival, modeled as a repeatability trait in a random regression model. Furthermore, the value of including CB data in addition to PB data in the genetic evaluation of longevity was investigated.

## MATERIALS AND METHODS

### Care and Use of Animals

Data recording and sample collection were conducted strictly in line with the legislation requirements in the 14 countries included in the study (Argentina, Belgium, Brazil, Canada, China, Germany, Iceland, Lithuania, the Netherlands, Poland, Russia, Spain, Sweden, and the United States). The data and samples collected for DNA extraction were obtained as part of routine data recording in the commercial breeding programs of Norsvin SA and Topigs Norsvin.

### Animals and Data

Data was available for two dam lines (breeds A and B) and their F1 cross (breed X; Table 1). Animals with available phenotypic information were born from 2013 to 2018 and had farrowed at least once. The animals were in commercial herds from 14 countries (see above), but not all countries had all breeds or combinations. Only data collected in farms with at least 50 animals and countries with at least 1,000 animals and with at least two breeds were included in the final data set. Six data sets were created with this data: breed A (A), breed B (B), and CBs (X) on their own, breed A with CBs (AX), breed B with CBs (BX), and all animals (ABX). For the latter three data sets, analyses where traits were considered different between breeds are indicated as A + X, B + X, and All, respectively.

**Table 1.** Number of animals with observations for each of the traits

Trait^*a*^	Breed A	Breed B	Crossbreds X
LGY12	31,735	209,198	53,861
LGY15	19,320	128,843	12,079
Survival	28,212	192,018	51,835

^*a*^Trait: LGY12, whether a sow was inseminated for a second litter within 85 d of first farrowing; LGY15, how many litters a sow had within 570 d of first farrowing (up to 5); Survival, repeatability trait indicating 0 if the sow survived to the next parity and 1 if she died after the current parity.

### Traits

Traits analyzed were two longevity traits already included in the breeding program of the PBs, and the new survival trait defined in this study. Longevity was defined as 1) whether or not (0 or 1) a sow was inseminated for a second litter within 85 d of first farrowing [Longevity 1–2 (LGY12)] and 2) how many litters she had within 570 d after the first farrowing [up to 5, Longevity 1–5 (LGY15)]. Survival was defined as the “probability of culling.” When animals did not reach the next parity, they were assigned a “1.” Otherwise, they were assigned a “0.” For example, a sow that produced a second litter (parity 2), but failed to produce a third litter, would have observation “0” for parity 1 and “1” for parity 2. However, if the sow is still alive at the end of parity 2, and may yet produce a third litter, she gets a “0” also for parity 2. Hence, if the sow does not have a “1” record, she is still alive and her longevity is censored (in the above case, she survived at least for two parities). This trait was analyzed with a repeatability model.

### Genotypes

Genotypes were available for 25,619 A, 25,692 B, and 1,994 F1 animals. All animals were genotyped using the Illumina GeneSeek custom 50K SNP chip (Lincoln, NE). Genome positions of the single-nucleotide polymorphism (SNP) were based on the SScrofa11.1 assembly of the reference genome (Groenen et al., 2012). Genotypes were filtered by excluding SNP with MAF <0.01 within population. Only SNP segregating in all three populations were kept, which resulted in 37,872 SNP after filtering.

### Statistical Analysis

Variance components were estimated using ASReml (Gilmour et al., 2009). For variance component estimation, only pedigree-based relationships (matrix **A**) were used, and the following model was applied:

$$\begin{array}{l} y=Xb+Zu+Vv+e, \end{array}$$

where **y** was a vector of observations (LGY12, LGY15, or Survival for 1, 2, or 3 breeds depending on data set); **X**, **Z**, and **V**, known incidence matrices; **b**, a vector of fixed effects (see below); **u**, a vector of random additive genetic effects, with **u** ~ N(0, **A**σ _u_^2^ or **H**σ _u_^2^), where σ _u_^2^ was the additive genetic variance; **v**, was a vector of common litter effects, with **v** ~ N(0, **I**_**v**_σ _v_^2^), where σ _v_^2^ was the variance of common litter effects; and **e**, a vector of residuals, with **e** ~ N(0, **I**_**e**_σ _e_^2^), where σ _e_^2^ was the residual variance. Matrices **I**_**v**_ and **I**_**e**_ were identity matrices of the appropriate dimensions, **A** was the additive relationship matrix (pedigree), and **H** was a matrix of combined pedigree and genomic relationships between individuals (Legarra et al., 2009; Christensen and Lund, 2010). Matrix **H** was built with calc_grm (Calus and Vandenplas, 2016) and estimation of breeding values was performed in MiXBLUP (Mulder et al., 2010) using the same model. Matrix **A** was only used for variance component estimation, while matrix **H** was used for breeding value estimation. Fixed effects were parity of dam, herd at first insemination, year of first insemination, and country for LGY12 and LGY15. For Survival, fixed effects were parity, parity of dam, herd year of insemination, country and season of insemination, and a fixed regression of number of weaned piglets on herd at farrowing. Survival is a repeatability trait. Normally, a permanent environmental effect is included in repeatability models. However, because of the nature of the data structure (i.e., only one potential outcome can be repeated), the permanent environmental variance (covariance between records of different parities) is very difficult to estimate (Ødegård et al., 2006). Attempts were made to estimate this effect and to fix this parameter at several different values to see what gave the best model fit. However, the best model fit was found when the permanent environmental effect was not included and, thus, we excluded it from the model. Other studies have also come to the same conclusion (Ødegård et al., 2006; Van Pelt et al., 2015). All traits were analyzed as both the same trait and as different traits between the breeds in separate analyses. Data sets A, B, X, AX, BX, and ABX were used when analyzing the traits as the same across populations, and “A + X”, “B + X,” and “All” were used when traits were analyzed as different between breeds. For validation of the models, PB animals born after January 1, 2015 and CB animals born after July 1, 2015 were masked. The reason for the difference in cutoff date between PBs and CBs was that, otherwise, only 200 CB would be in the training set, which is too few animals. Among the masked animals, the 5,000 youngest animals within breed with an observation for LGY15 were used for validation (Table 2). Among the masked animals, there were too few animals that had both genotypes and phenotypes, so validation animals were a mix of genotyped and ungenotyped animals.

**Table 2.** Number of animals in training and validation sets

	Breed A		Breed B		Crossbreds X	
Trait^*a*^	Training	Validation	Training	Validation	Training	Validation
LGY12	10,642	5,000	72,135	5,000	2,257	5,000
LGY15	10,642	5,000	72,135	5,000	2,257	5,000
Survival	9,601	5,000	69,705	5,000	2,245	5,000

^*a*^Trait: LGY12, whether a sow was inseminated for a second litter within 85 d of first farrowing; LGY15, how many litters a sow had within 570 d of first farrowing (up to 5); Survival, repeatability trait indicating 0 if the sow survived to the next parity and 1 if she died after the current parity.

Models were compared for the accuracy of estimated breeding values (EBVs):

$$\begin{array}{l} \frac{cor(CP,\;\;\;EBV)}{\sqrt{h^{2}}}, \end{array}$$

where CP was the corrected phenotype (phenotype corrected for fixed and nongenetic random effects) of LGY12, LGY15, or Survival (as calculated by MiXBLUP when all animals were in the data set), EBV was the breeding value of each model (when validation animals were not in the training set), and *h*^2^ was the heritability of LGY12, LGY15, or Survival when all breeds were in the data set. The reason for correlating with the CP of all models was to compare the model results against each other (because a single gold standard was not available). However, using phenotypes from LGY15 to predict LGY12 is not really of interest because the LGY15 phenotype is recorded later in life than the LGY12 phenotype; thus, these results are not presented. This is to some extent also true for Survival, which will be similar to LGY12 until the third parity (LGY12 is only recorded up until the second parity). That is, Survival is recorded from the first parity onward, whereas LGY12 is recorded at the onset of the second parity. As all the sows in this study had already farrowed once, predicting LGY12 with LGY15 or Survival phenotypes is not really interesting. However, comparing performance of LGY12 and Survival with each other when predicting other traits is interesting because both can be measured early in life.

In addition, the correlation between EBVs when using the full data set for the validation animals (for each breed) were compared to using information (phenotypes) also from other breeds. The correlation between EBVs for the different traits (within breed) were also compared. Because Survival is a repeatability trait, and LGY12 and LGY15 are not, the heritability estimates are not directly comparable. Therefore, an additional equation was used to estimate the heritability of the mean of *n* survival records, *h*_*n*_^2^:

$$\begin{array}{l} {h_{n}}^{2}=\frac{\sigma_{g}^{2}}{{}^{\sigma_{e}^{2}}╱{}_{n}+\;\;\;\sigma_{g}^{2}+\;\;\;\sigma_{v}^{2}\;\;\;}, \end{array}$$

where σ _g_^2^ was the additive genetic variance, σ _e_^2^ was the residual variance, σ _v_^2^ was the variance of common litter effects, and *n* was the average number of parities within breed.

## RESULTS

Descriptive statistics are in Table 3. The mean number of parities in the survival data set were 2.95, 3.97, and 3.37 for breed A, breed B, and CBs, respectively. Maximum number of parities in the survival data set were 10, 14, and 9 for breed A, breed B and CBs, respectively. The lower maximum number of parities in CB could be because the first CB to be inseminated were inseminated a year later than the first PB and, thus, did not have the opportunity to have as many litters. Plotting the proportion of animals for each maximum parity within breed resulted in a similar curve across breeds (result not shown). Proportionally, 4.23% and 14.67% of sows had more than seven parities in breeds A and B, respectively. Favoring first-parity gilts to speed up genetic progress may affect the proportion of sows at higher parities.

**Table 3.** Descriptive statistics

Trait^*a*^	Breed	Mean	SD	Min.	Max.
LGY12	Breed A	0.83	0.37	0	1
	Breed B	0.90	0.30	0	1
	Crossbreds X	0.91	0.28	0	1
LGY15	Breed A	3.17	1.61	1	5
	Breed B	3.82	1.40	1	5
	Crossbreds X	4.05	1.34	1	5
Survival	Breed A	0.22	0.42	0	1
	Breed B	0.12	0.33	0	1
	Crossbreds X	0.07	0.25	0	1

^*a*^Trait: LGY12, whether a sow was inseminated for a second litter within 85 d of first farrowing; LGY15, how many litters a sow had within 570 d of first farrowing (up to 5); Survival, repeatability trait indicating 0 if the sow survived to the next parity and 1 if she died after the current parity (lower means are favorable).

### Heritability and Genetic Correlations

Heritability estimates (Table 4) were similar across data sets when traits in PBs and CBs were considered as the same trait, with the exception of data set X for LGY15. Slightly greater heritabilities for LGY15 and Survival were observed for breed B compared to breed A when these were analyzed separately, but breed A had a greater heritability than breed B for LGY12. The adjusted heritability estimates (*h*_*n*_^2^) for Survival were 0.048, 0.094, and 0.040 for A, B, and X, respectively, when only one breed was in the data set.

**Table 4.** Heritabilities (SE) when traits^*a*^ were considered the same across breeds

Data set^*b*^	LGY12	LGY15	Survival
A	0.074 (0.009)	0.096 (0.013)	0.017 (0.003)
B	0.063 (0.004)	0.132 (0.007)	0.026 (0.001)
X	0.073 (0.009)	0.049 (0.015)	0.013 (0.002)
AX	0.066 (0.006)	0.089 (0.010)	0.017 (0.002)
BX	0.066 (0.004)	0.128 (0.006)	0.026 (0.001)
ABX	0.083 (0.004)	0.125 (0.006)	0.025 (0.001)

^*a*^Trait: LGY12, whether a sow was inseminated for a second litter within 85 d of first farrowing; LGY15, how many litters a sow had within 570 d of first farrowing (up to 5); Survival, repeatability trait indicating 0 if the sow survived to the next parity and 1 if she died after the current parity.

^*b*^Data set: A, breed A; B, breed B; X, F1 crossbred of A and B; AX, A and X animals; BX, B and X animals; ABX, all animals (all three breeds).

When traits were considered different between breeds, larger differences between heritability estimates between the breeds were observed, especially for LGY15 (Table 5). For LGY15, CB heritabilities increased when breed B was included. This effect was not seen when CB were combined with breed A. For LGY12, heritability estimates for breed A decreased when CB were in the data set (as opposed to only including breed A animals) but not when all three breeds were in the data set. This effect was not seen for breed B. For CB, the heritability estimates for LGY12 did not change depending on whether other breeds were included or not. Genetic correlations between breed A and the CBs were low and not statistically significant from 0 for LGY12 or LGY15. When all breeds were in the data set, this correlation decreased even further. Between breed B and the CBs, the genetic correlation was statistically significant from 0 only for LGY15. For Survival, variance component estimation by Resticted Maximum Likelihood (REML) did not converge when Survival was considered as different traits for each of the breeds and, therefore, results are not included.

**Table 5.** Heritabilities (SE) and genetic correlations (SE) when traits were considered as different between breeds

		Heritabilities (SE)			Correlations (SE)	
Trait^*a*^	Data set^*b*^	Breed A	Breed B	Crossbreds X	A–X	B–X
LGY12	A + X	0.045 (0.007)	—	0.072 (0.009)	0.308 (0.203)	—
	B + X	—	0.061 (0.004)	0.079 (0.009)	—	0.231 (0.115)
	All	0.073 (0.009)	0.061 (0.004)	0.079 (0.009)	0.128 (0.193)	0.231 (0.116)
LGY15	A + X	0.095 (0.013)	—	0.048 (0.015)	0.485 (0.342)	—
	B + X	—	0.130 (0.007)	0.064 (0.017)	—	0.614 (0.187)
	All	0.096 (0.013)	0.130 (0.007)	0.064 (0.017)	0.245 (0.336)	0.610 (0.187)

^*a*^Trait: LGY12, whether a sow was inseminated for a second litter within 85 d of first farrowing; LGY15, how many litters a sow had within 570 d of first farrowing (up to 5).

^*b*^Data set: A + X = breed A and CB animals; B + X, breed B and CB animals; All, all animals (A, B, and X).

### Prediction Accuracy

Prediction accuracies when traits were considered the same across breeds varied a lot between PB and CB animals (Table 6–8). Predicting LGY12 was generally poor (<0.2) but was greater for predicting CB than PB (Table 6). Overall, SEs were high in relation to estimates. The greatest accuracy was found for LGY12 for CBs when not all breeds were in the data set. Prediction accuracy of CBs had a tendency to be greater when CBs were analyzed with breed B compared to with breed A. Both PB lines benefitted from including CB data but not the other PB line.

**Table 6.** Prediction accuracies (SE) for LGY12^*a*^ when traits were considered the same across breeds

	Breed		
Data set^*b*^	A	B	X
Single breed	0.068 (0.049)	0.036 (0.049)	0.135 (0.050)
AX	0.095 (0.049)	—	0.179 (0.050)
BX	—	0.077 (0.049)	0.130 (0.050)
ABX	0.086 (0.049)	0.019 (0.049)	0.053 (0.050)

^*a*^Trait: LGY12, whether a sow was inseminated for a second litter within 85 d of first farrowing.

^*b*^Data set: Single breed, only one breed per data set (A, B, or X); AX, breed A and CB animals; BX, breed B and CB animals; ABX, all animals (all three breeds).

**Table 7.** Prediction accuracies (SE) when traits were considered the same across breeds and gold standard was CP_LGY15_

		Breed		
Trait^*a*^	Data set^*b*^	A	B	X
LGY12	Single breed	0.125 (0.040)	0.122 (0.040)	0.033 (0.041)
	AX	0.138 (0.040)	—	0.023 (0.041)
	BX	—	0.105 (0.040)	0.099 (0.040)
	ABX	0.155 (0.040)	0.083 (0.040)	0.019 (0.041)
LGY15	Single breed	0.268 (0.040)	0.286 (0.040)	0.032 (0.041)
	AX	0.275 (0.040)	—	0.011 (0.041)
	BX	—	0.269 (0.040)	0.120 (0.040)
	ABX	0.289 (0.040)	0.185 (0.040)	0.061 (0.041)
Survival	Single breed	0.204 (0.040)	0.290 (0.040)	0.003 (0.041)
	AX	0.208 (0.040)	—	0.015 (0.041)
	BX	—	0.281 (0.040)	0.112 (0.040)
	ABX	0.196 (0.040)	0.213 (0.040)	0.038 (0.040)

^*a*^Trait: LGY12, whether a sow was inseminated for a second litter within 85 d of first farrowing; LGY15, how many litters a sow had within 570 d of first farrowing (up to 5); Survival, repeatability trait indicating 0 if the sow survived to the next parity and 1 if she died after the current parity.

^*b*^Data set: Single breed, only one breed per data set (A, B, or X); AX, breed A and CB animals; BX, breed B and CB animals; ABX, all animals (all three breeds).

**Table 8.** Prediction accuracies (SE) when traits were considered the same across breeds and gold standard was CP_Survival_

		Breed		
Trait^*a*^	Data set^*b*^	A	B	X
LGY12	Single breed	0.125 (0.098)	0.168 (0.094)	0.019 (0.094)
	AX	0.178 (0.098)	—	0.064 (0.094)
	BX	—	0.177 (0.094)	0.183 (0.094)
	ABX	0.230 (0.098)	0.035 (0.094)	0.157 (0.094)
LGY15	Single breed	0.308 (0.098)	0.338 (0.094)	0.093 (0.094)
	AX	0.340 (0.098)	—	0.042 (0.094)
	BX	—	0.261 (0.094)	0.108 (0.094)
	ABX	0.375 (0.098)	0.086 (0.094)	0.096 (0.094)
Survival	Single breed	0.169 (0.098)	0.547 (0.094)	0.092 (0.094)
	AX	0.189 (0.098)	—	0.002 (0.094)
	BX	—	0.426 (0.094)	0.062 (0.094)
	ABX	0.267 (0.098)	0.310 (0.094)	0.033 (0.094)

^*a*^Trait: LGY12, whether a sow was inseminated for a second litter within 85 d of first farrowing; LGY15, how many litters a sow had within 570 d of first farrowing (up to 5); Survival, repeatability trait indicating 0 if the sow survived to the next parity and 1 if she died after the current parity.

^*b*^Data set: Single breed, only one breed per data set (A, B, or X); AX, breed A and CB animals; BX, breed B and CB animals; ABX, all animals (all three breeds).

When using CP_LGY15_ as gold standard, the prediction accuracies for PB animals were similar between A and B animals, although there was a tendency for breed B to have greater predictive ability for Survival than breed A (Table 7). Crossbred animals had poor prediction accuracy, and SEs were high compared to the estimates except for when they were analyzed with breed B animals. For breed B, prediction accuracy decreased when CBs were in the data set, while, for breed A animals, the tendency was that prediction accuracy increased when CBs were included in the data set. For breed A, prediction accuracy for the Survival trait tended to be lower than for LGY15, whereas, for breed B, the opposite was seen.

When using CP_Survival_ as the gold standard (Table 8), prediction accuracies for breed A tended to be greater for LGY15 than for Survival, while the opposite was seen for breed B. Again, breed A seemed to benefit from including CB phenotypes, while breed B did not. Prediction accuracies for CBs were poor, except when analyzed together with breed B for the LGY traits. In fact, for LGY12, CB tended to have a greater prediction accuracy than breed B for data sets “BX” and “ABX.”

When predicting LGY12 and the trait was considered different between breeds, most of the estimates were smaller than their SE (Table 9). The greatest prediction accuracy was for CB when these were analyzed together with breed A.

**Table 9.** Prediction accuracies (SE) for LGY12^*a*^ when traits were considered different across breeds

	Breed		
Dataset^*b*^	A	B	X
A + X	0.070 (0.049)	—	0.206 (0.050)
B + X	—	0.036 (0.049)	0.024 (0.050)
All	0.070 (0.049)	0.002 (0.049)	0.086 (0.050)

^*a*^Trait: LGY12, whether a sow was inseminated for a second litter within 85 d of first farrowing.

^*b*^Data set: A + X, breed A and CB animals; B + X, breed B and CB animals; All, all animals (all three breeds).

When traits were considered different between the breeds, and the gold standard was CP_LGY15_, prediction accuracies for CB were poor, and had high SEs (Table 10). For both of the PB (A and B), prediction accuracies were similar to those obtained when traits were considered the same between breeds.

**Table 10.** Prediction accuracies (SE) when traits were considered different between breeds and gold standard was CP_LGY15_

		Breed		
Trait^*a*^	Data set^*b*^	A	B	X
LGY12	A + X	0.129 (0.040)	—	0.056 (0.041)
	B + X	—	0.121 (0.040)	0.012 (0.041)
	All	0.127 (0.040)	0.131 (0.040)	0.006 (0.040)
LGY15	A + X	0.271 (0.040)	—	0.019 (0.041)
	B + X	—	0.283 (0.040)	0.070 (0.040)
	All	0.270 (0.040)	0.284 (0.040)	0.047 (0.040)

^*a*^Trait: LGY12, whether a sow was inseminated for a second litter within 85 d of first farrowing; LGY15, how many litters a sow had within 570 d of first farrowing (up to 5).

^b^Data set: A + X, breed A and CB animals; B + X, breed B and CB animals; All, all animals (all three breeds).

Correlations between EBVs of different traits within breed were similar between PBs (Supplementary Table S1). For PBs (within breed), the greatest correlations were found between LGY15 and Survival (−0.797 and −0.720 for breeds A and B, respectively), followed by LGY12 and LGY15 (0.610 and 0.690 for breeds A and B, respectively). Correlations were lower in CB and, here, the greatest correlation was between LGY12 and LGY15 (0.495), although the correlation between LGY15 and Survival was very similar (0.434).

Correlations between breeding values for PB animals using different sources of information were calculated (Supplementary Table S2). The correlation between EBVs for PB animals when estimated using only CB data compared to when PB data was included were very low (<0.3). When the PB phenotypes were included in the data set together with other breeds (AX, BX, or All), most correlations were >0.9.

Correlations between breeding values for CB based on CB data and other sources of information were also calculated (Supplementary Table S3). Correlations were very low when CBs were not included in the data set (mostly <0.1). When CBs were included, correlations ranged from 0.54 to 0.97 across traits, with the greatest correlations for LGY15. Generally, the correlation was greater when CBs were combined with breed A (AX) than with breed B (BX).

When traits are analyzed as different traits between the breeds, both PB and CB get EBVs for both traits. Thus, it is possible to get an estimate of the correlation between PB and CB performance based on EBVs. For PBs there was a high correlation between PB and CB performance for LGY15 (>0.9; Supplementary Table S4). When analyzed with breed B, this correlation was also high for CB (0.90). For LGY12, breed A and CBs had similar correlations (~0.7), whereas for breed B and CB the difference was larger (0.64 and 0.45, respectively).

**Figure 1.**
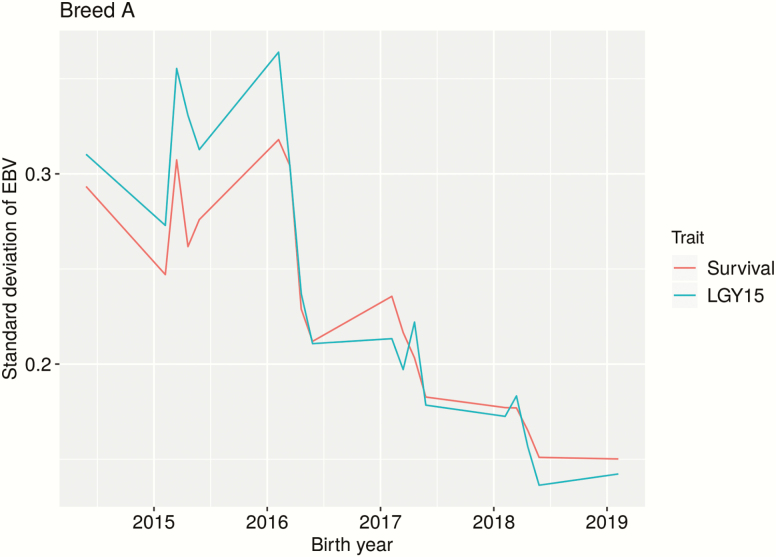
Standard deviation of EBVs (scaled by the genetic SD) for genotyped animals of breed A across birth years, using only purebred data. Traits: Survival is a repeatability trait indicating if the sow survived to the next parity; LGY15 is the number of parities a sow has within 570 d of first farrowing (up to 5).

**Figure 2.**
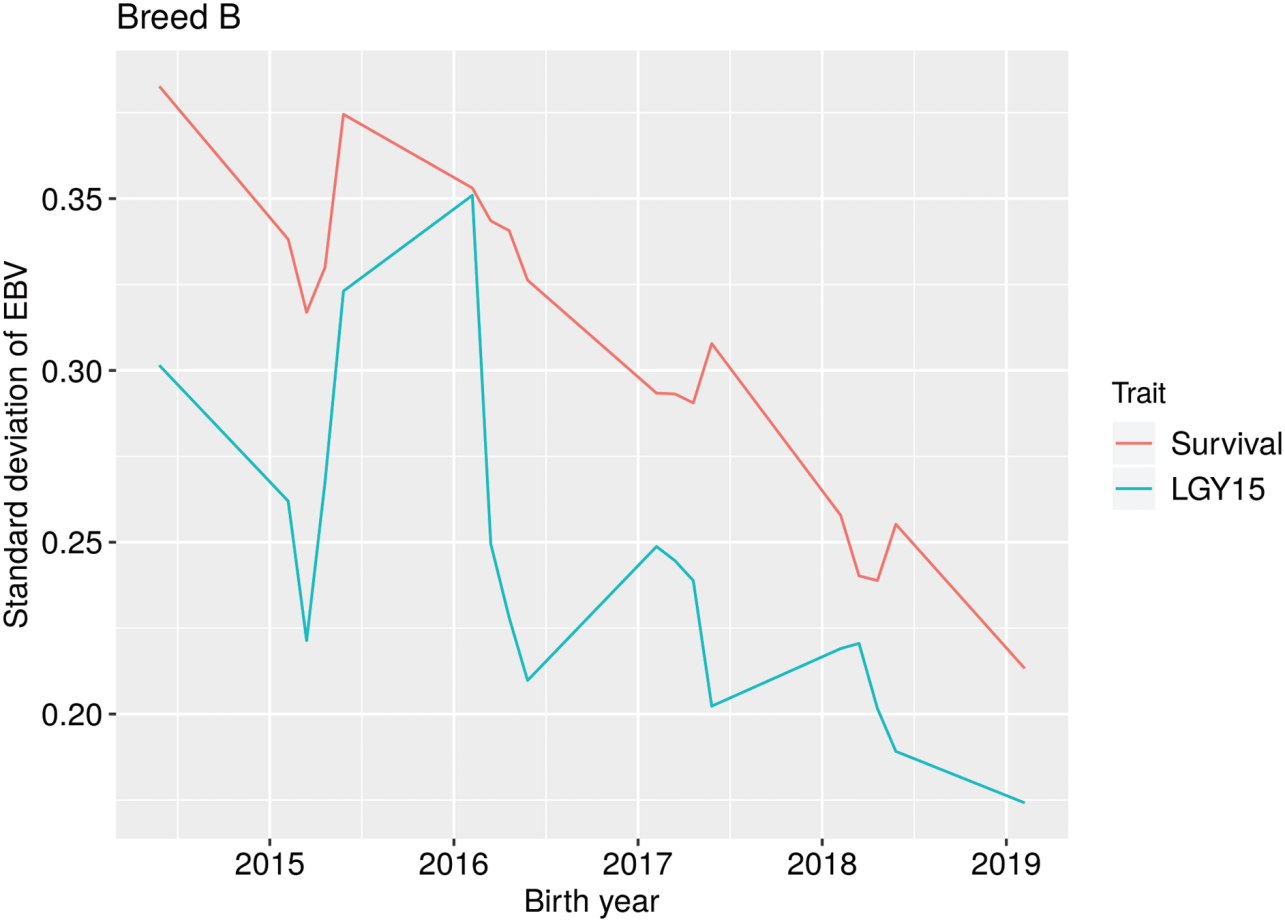
Standard deviation of EBVs (scaled by the genetic SD) for genotyped animals of breed B across birth years, using only purebred data. Traits: Survival is a repeatability trait indicating if the sow survived to the next parity; LGY15 is the number of parities a sow has within 570 d of first farrowing (up to 5).

By plotting the SD of EBVs (scaled by the genetic SD) across birth years (for genotyped animals), it is possible to get an idea of when information is available for different animals. Here, the assumption is that greater SD equals greater accuracy. Big differences were seen between the PBs (Figs 1 and 2). Reading the graphs from right to left (i.e., from 2019), it was seen that EBV accuracy increases over time (i.e., the older the animal, the higher the accuracy). For breed B (Fig. 2), especially for young animals, Survival gave greater accuracy than LGY15. When animals are about 3 years of age (born 2016), they get their own phenotype for LGY15, and the two traits appear about equal. There is an unexpected dip in accuracy for animals born in early 2015, though there seems to be a dip mid-year every year. For breed A, the accuracy for both traits seemed equal for young animals, and a real difference was only seen after animals get their own phenotype for LGY15 (at around 3 years of age).

Based on the solutions for the fixed effects for the Survival trait, it seemed that it was somewhat harder for breed B sows inseminated in season 4 (i.e., October–December) to produce another litter (Supplementary Table S5). This was not seen for the other breeds and the effect was small. Breed A sows were less likely to survive after parity 3, whereas this cutoff was parity 5 for breed B and CB. There was no difference in Survival for breed A and CBs based on the parity of the dam of the sow, but breed B sows were more likely to survive if parity of dam was >1. There were differences between countries, and this was consistent between breeds, suggesting that countries differ in rearing conditions and/or culling decisions. Larger fixed effects indicate that it was harder to survive. Grouped fixed effects for countries were: >0.2 for Lithuania and the United States; >0.1 for Argentina, Brazil, and Germany; between 0.0–0.1 for China, Russia, Canada, Sweden, Spain, the Netherlands, and Belgium; and <0.0 (i.e., negative value) for Poland and Iceland.

## DISCUSSION

This study investigated the contribution of CB data in addition to PB data on the estimation of breeding values for longevity in pigs. Furthermore, the possibility of the use of an alternative definition of longevity, here called Survival, was investigated. The results were not univocal but indicated that adding CB information in the genetic evaluation is important for improving longevity in CBs.

### Genetic Parameters

Heritability estimates of LGY12 and LGY15 ranged from 0.063 to 0.132 depending on which breeds were included in the data set. For the Survival trait, heritability estimates ranged from 0.017 to 0.026. Because this is a repeatability trait, the heritability is expected to be lower than for LGY12 and LGY15. The reason for this is that the heritability is estimated per parity, not over the whole life. However, adjusted heritability (*h*_*n*_^2^ = 0.049–0.094) was also lower than for LGY15. Heritability estimates vary with the definition of the trait and method of analysis. Other studies have found heritability estimates of longevity using a linear model in the range 0.03–0.10 (Serenius and Stalder, 2004; Engblom et al., 2009), while, with survival analysis models, in the range of 0.11–0.31 (Serenius and Stalder, 2006). The linear estimates for longevity from other studies (see above) were similar to our estimates for LGY12 and LGY15. For productive life using survival analysis models, heritability estimates have been found in the range 0.06–0.19 (Serenius and Stalder, 2004; Casellas et al., 2008; Engblom et al., 2009), while, when analyzed with a linear model, they were in the range of 0.17–0.25 (Guo et al., 2001; Serenius et al., 2008). Heritability estimates of stayability, that is, ability to stay until the next parity, have been found in the range 0.02–0.27 (Stalder et al., 2004; Serenius and Stalder, 2006; Aasmundstad et al., 2014). As in the current study, Engblom et al. (2009) also found different heritability estimates for different breeds.

Genetic correlations between PB and CB longevity (LGY12 and LGY15) were low, suggesting that PB and CB longevity are different traits similar to Abell et al. (2016). One reason why PB and CB performance may differ for a trait is due to genotype by environment interaction (G × E; Wientjes and Calus, 2017). This is not unlikely, considering that PB and CB are usually kept in different environments, where handling and culling decisions may be different. Reasons for culling are often also very varied and may not be the same in PB and CB populations. The reason for culling that is most often stated is reproductive failure, closely followed by leg problems (Stalder et al., 2004). Furthermore, the reasons for reproductive failure are also complex and often coinciding and may be caused by many different factors, such as pain, incorrect feeding (too much/little), and management. Thus, the genetic aspect of longevity is hard to disentangle from more external effects on longevity (Vukasinovic, 1999). Based on the genetic correlations, it seems that PB longevity may not be a good predictor for CB longevity. Therefore, CB data is needed if the goal is to improve CB longevity.

### Prediction Accuracies

Using different measures of longevity yield deviations as gold standard can give an indication as to whether phenotypes from one trait are able to predict alternative measures/aspects of longevity. For example, using CP_LGY15_ as gold standard indicates whether Survival can predict LGY15, which is the trait Topigs Norsvin uses today. For the two traits available early in life (LGY12 and Survival), Survival tended to have a greater prediction accuracy than LGY12 both when predicting CP_LGY15_ and CP_Survival_.

For CP_LGY15_ and CP_Survival_, the same trend was seen in both cases when traits were considered the same across breeds. For breed A, LGY15 tended to give the greatest prediction accuracy (within breed), whereas, for breed B, Survival tended to give the greatest prediction accuracy. However, within breed, when predicting CP_LGY15_, the prediction accuracies were very similar between the two traits. This suggests that both LGY15 and Survival can predict LGY15 to the same accuracy. As Survival is available earlier in life, it is advantageous to use Survival. In both cases, it also seems that breed A benefited from including CB data, while, for breed B, this was not the case. This may be due to the number of phenotypes for each breed. Breed B had many more phenotypes than breed A, so CB would have contributed more to the data set with breed A when these were included (and traits were considered the same across breeds). As the number of CB phenotypes were close to the number of phenotypes from breed A, when considering the traits as the same between PB and CB, CB phenotypes clearly contributed to breed A breeding values. For breed B, probably there were so many within-breed phenotypes that CB did not contribute to improve prediction of breed B breeding values. In addition, the genetic correlation between CB and the PB differed between breed A and breed B. It was greater between CB and breed B than between CB and breed A, suggesting that CB phenotypes would contribute more for breed B evaluations. As the genetic correlation was not very high (considerably less than 1), this suggests that longevity is a different trait in PB and CB, which would reduce accuracies when the traits are considered the same across breeds. In addition, most of the CB had breed A sires, so the relationship between animals with phenotypes between breed A and CB would have been lower than it was between breed B and CB.

For CB animals, prediction accuracy was very poor, regardless of the trait definition or gold standard. There are several possible reasons for this. One is that the training set was relatively small and that ~61% of animals in the training set had score 5 for LGY15 (i.e., five parities). Thus, there was little variation in the training and validation sets. In addition, especially for data set X (only CB), no parents had phenotypes, meaning that full and/or half-sibs would have been the closest relatives. Again, because of the low genetic correlation, even when PB animals were included in the training data set (i.e., AX, BX, and ABX), PB information may have contributed very little as longevity does not seem to be the same trait between PB and CB.

When predicting LGY12 and the trait was considered different between breeds, analyzing the PB with or without CB gave very similar prediction accuracies. This suggests that CB phenotypes do not really contribute to prediction accuracies of PB for PB performance. The low genetic correlation between PB and CB for LGY12 supports this. In addition, most PB sows would have their own phenotype for LGY12 before any CB offspring get the phenotype. Analyzing CB data together with breed A was favorable for prediction of CB compared to analyzing CB alone, whereas this was unfavorable when analyzing with breed B for LGY12. For gold standard CP_LGY15_, the same was seen for both the PB and the CB as for CP_LGY12_. For EBV_LGY15_, CB benefitted from being analyzed with breed B but not with breed A. As the genetic correlation between breed B and CB were greater than for breed A and CB, this makes sense.

Prediction accuracies in the current study were in the range 0.003–0.375, 0.001–0.547, and 0.003–0.183 for breed A, breed B, and CB, respectively, depending on trait and gold standard. Few other studies have reported prediction accuracy of longevity traits, but Aasmundstad et al. (2015) found prediction accuracies for stayability in the range 0.31–0.55 depending on validation animals and whether or not genomic information was used. This is similar to the greatest prediction accuracies for PB in the current study.

Based on the SD of EBVs over birth year (see Figs 1 and 2), it seemed that Survival is a better trait than LGY15 for breed B but not for breed A. For breed B, it was evident that Survival was especially beneficial for young animals. This makes sense as animals get a phenotype earlier in life and that parents and older relatives have multiple observations (for Survival). Once animals are old enough to have their own phenotype for LGY15 (born 2016 or earlier), this evens out. The steady increase in accuracy as animals get older is due to more data being available as older relatives get phenotypes. As these graphs only show genotyped animals, there were relatively few animals born before 2016, which may explain the dip in accuracy for animals born before this. In addition, most animals born before 2016 did not have parents with phenotypes in the data set. However, a similar pattern was seen when all animals were included, except with a less pronounced dip for animals born before 2016 (results not shown). While the number of animals with genotypes were relatively evenly distributed between breed A and B, breed B had more genotyped animals with phenotypes (~8,000 compared to ~3,700 for breed A). In addition, breed B had more phenotypes in general and, thus, more relatives with data, which increases the accuracy of EBVs. This may explain why there was a clearer pattern for breed B than for breed A as there would have been more power to detect a pattern. This suggests that a lot of data is needed to evaluate longevity traits.

### Similarities of Traits

When looking at the EBVs for the different traits within breed, the greatest correlation between traits was between LGY15 and Survival for PB. This is not surprising as these traits are supposed to measure the same thing, only with different definitions. The advantage of the Survival trait is, however, that the first observation is available much earlier in life than LGY15, which should give more accurate breeding values earlier in life. This was not seen for CB, where the greatest correlation was seen between LGY12 and LGY15. However, the correlation between LGY15 and Survival was a close second. Why LGY15 should be more closely correlated to LGY12 than Survival is unclear but could be because Survival has the potential to have more outcomes than LGY15 and, thus, LGY15 is, therefore, more closely related to LGY12. In addition, only ~15% of the CB had two or fewer parities, and 60% of the CB had LGY15 = 5, so these two traits would be very closely related in the CB and could explain this difference between PB and CB. The lowest correlation was between LGY12 and Survival for all breeds. What is clear from all these correlations, however, is that regardless of the trait used in the breeding program, selecting the best animals based on the EBV would move the traits in the same direction (as high values of Survival equal low values for LGY15). However, the speed of genetic progress would differ based on the trait chosen and at which age selection takes place.

### Environmental Factors

The fixed effects results indicate that there exists a seasonal effect of Survival for breed B. A lower survival rate seems to be achieved when the sows were inseminated in season 4 (October–December). There is some evidence for more culling in certain seasons, mainly due to light or temperature (Stalder et al., 2004). However, it is unlikely that this has a major effect in modern facilities with light and temperature regulation. Most, but not all of the herds in this study were housed in modern facilities, but about 14% of breed B animals (and 1.7% of breed A animals) were in herds without temperature regulations. This could explain why a small effect of season was seen for breed B. This effect was, however, very small: 10% chance of culling compared to 8 or 9%.

It seemed that there was a higher replacement rate in breed A than in breed B and CB (based on average number of parities). Some studies have found differences between nucleus and commercial herds, where culling decisions are different (Aasmundstad et al., 2014). Aasmundstad et al. (2014) found that when including EBV at the time of removal in the prediction model (to account for culling based on EBV), heritability estimates of stayability were greater when using data from nucleus herds. In the current study, we included number of weaned piglets within herd of farrowing as a regression to account for performance as a criterion for culling. However, as nucleus herds were excluded in this study, that is likely not the cause of the different number of parities here. Ideally, culling reasons should be routinely recorded so that it is possible to distinguish between voluntary (i.e., for performance) or involuntary culling (i.e., health or reproductive problems). As these were not available, it is not possible to conclude whether differences in culling between PBs were due to genetic or other reasons. Differences in survival between countries were found as well but, if a country had poor survival, this affected the survival for all of the breeds. Differences between countries can be due to many different factors, such as housing conditions, herd type, management system (including culling decision), type of housing (e.g., loose housing or fixation of sows), feed type, and temperature. As there seems to be some G × E between PB and CB, it is likely that this is also the case between countries. It may be of interest to analyze longevity or survival traits as different between countries, or between different housing systems, to determine whether the traits are genetically the same across environments. Several studies have found differences in longevity in different environments or housing systems (Morris et al., 1998; Stalder et al., 2004), but these have not looked at genetic effects of the trait. In Drosophila, G × E effects have been found for longevity (Vieira et al., 2000), so this might be present also in other species.

Between-breed differences were observed for the effect of parity of dam. In breed B, if the dam’s parity was greater than 1, it was easier for the sow to survive until the next parity. Thus, if the dam had proven that she could have more than one litter, this makes it more likely that her offspring (sows) will too. The same effect was not seen in breed A. It seems the dam effect was less strong in breed A or that culling decisions were based on different criteria. For CB, no effect of parity of dam was seen, perhaps indicating that CBs are kept until the next parity independent of breeding value of parents. This would result in a small heritability as the genetic potential of the animal (the EBV) is not taken into account and, thus, the genetic aspect has little impact on whether the animal stays for another parity. Therefore, CB will mainly be kept based on their own performance in the previous litter rather than the performance of their parents or EBV. This agrees with the low genetic correlation between PB and CB longevity, where dam longevity was not indicative of CB longevity (Abell et al., 2016).

### Model Considerations

One of the challenges with longevity traits is that the trait is collected late in life, or after the animal is dead, such as length of productive life (Vollema and Groen, 1998; Vukasinovic, 1999; Hoge and Bates, 2011). For animals that are still alive, or too young to have the phenotype, observations are censored. The problem with censored data is that it creates bias both if it is excluded and if it is included. The former because not all available information is used (long longevity records are excluded) and the latter because it gives an incorrect picture of actual phenotypes (for animals that are still alive, longevity is underestimated). Linear models cannot include censored data but survival analysis models can (Vukasinovic, 1999). However, the latter require analysis with a specialized software (e.g., “Survival Kit”; Ducrocq and Sölkner 1998) before including the trait in breeding value estimations. In addition, it is not possible to use in a multitrait model with other linear traits, which is common in most breeding value estimations (Guo et al., 2001; Serenius and Stalder, 2006). However, Meuwissen et al. (2002) found very similar correlations between true and predicted breeding value for linear, binary, and proportional hazard models in simulated data. The advantage of the new Survival trait in this study is that observations can be used as soon as they are available, and they are available much earlier in life than LGY15. This should alleviate the problem with censored data as survival is recorded at each parity of the sow. An observation on LGY15 may only be available after descendants are already selected, which is too late. In addition, culling criteria may vary over time (Vukasinovic, 1999), and it is not possible to take this into account for LGY15 because there is only one observation, whereas, for a repeatability trait, such as Survival, time-dependent herd effects (which to some extent includes culling criteria) can be included.

One disadvantage of the Survival trait in the current study was that the variance components could not be estimated when the traits were considered different between the breeds. This may be due to the binary nature (0–1) of the trait so that genetic (co)variance estimates are rather low. It is also possible that the prediction model needs some adjustment depending on which breeds, or possibly herds, are included. It is possible that the model should not be the same for breed A with CB and breed B with CB. However, as we do not have an estimate of genetic correlations between PB and CB for the Survival trait, it is not possible to conclude whether Survival is the same trait in PB and CB. It is likely that this genetic correlation between PB and CB for the Survival trait would be similar to what was found for LGY15.

An advantage of the Survival trait is that its recording pattern fits well with the routine evaluations for maternal traits, which are mostly collected per parity, for example, total number born, litter weight, and body condition score of the sow. Further research is needed to investigate the correlations between Survival and these maternal traits. As litter size seems to have a large effect on whether or not an animal is culled (Yazdi et al., 2000; Guo et al., 2001; Hoge and Bates, 2011), a multitrait analysis seems needed for routine evaluations. However, number of piglets weaned was in the prediction model for Survival in the current study, so its effect was accounted for, although in a different manner than in a multitrait model.

The analysis of the Survival trait using a repeatability model assumes a correlation between parities of 1, which is not strictly correct (Madgwick and Goddard, 1989). An alternative is to treat Survival as a different trait between parities, but this may be too computationally demanding and, thus, assuming a correlation of 1 may be optimal in terms of computational costs (Madgwick and Goddard, 1989). An attempt was made in the current study to analyze Survival as a different trait between parities but estimates of variance components did not converge. An alternative would be to use a random regression model (Mrode, 2014). Random regression models have been shown to be robust to censoring, and time-dependent variables can be included to account for different environmental and genetic influences over time while also being easier to implement than survival analysis models (Veerkamp et al., 1999).

## CONCLUSIONS

Results from this study show that longevity traits of PB and CB animals are not genetically the same trait. Thus, it is necessary to collect data in CB populations if the goal is to improve PB breeds for CB longevity. Prediction accuracies for LGY15 and the new Survival trait were similar. As Survival is available earlier in life, this trait would be preferred. This pattern was evident in breed B, but less so in breed A. More research and data will be needed to estimate genetic correlations between PB and CB survival. It is clear from this study that a large amount of data is needed to get accurate predictions of longevity, both in PBs and CBs.

## Supplementary Material

txaa073_suppl_Supplementary_Tables
