# Supplementary material for: Predicting survival and longevity of sows using purebred and crossbred data
Source: Transl Anim Sci. 2020 Jun 1;4(2):993–1005. doi: 10.1093/tas/txaa073 (PMC7299294; doi:10.1093/tas/txaa073)
Supplement: txaa073_suppl_Supplementary_Tables [file txaa073_suppl_supplementary_tables.docx]

**Supplementary file 1**

**Table S1**. Correlations (SE) between EBVs between traits^1^ within breed

| Trait 1 | Trait 2 | Breed A | Breed B | Crossbred X |
| --- | --- | --- | --- | --- |
| LGY12 | LGY15 | 0.610 (0.011) | 0.690 (0.010) | 0.495 (0.012) |
| LGY12 | Survival | -0.500 (0.012) | -0.627 (0.011) | -0.226 (0.014) |
| LGY15 | Survival | -0.797 (0.008) | -0.720 (0.009) | -0.434 (0.013) |

^1^Trait: LGY12 = whether a sow was inseminated for a second litter within 85 days of first farrowing, LGY15 = how many litters a sow had within 570 days of first farrowing (up to 5), Survival = repeatability trait indicating 0 if the sow survived to the next parity and 1 if she died after the current parity.

**Table S2**. Correlations between estimated breeding values for purebred validation animals using the full dataset. Correlations are between estimated breeding values for purebred animals when they were analyzed alone compared to using other sources of information

|  | Breed A | | | Breed B | | |
| --- | --- | --- | --- | --- | --- | --- |
| Dataset^1^ | LGY12^2^ | LGY15 | Survival | LGY12 | LGY15 | Survival |
| X | 0.267 | 0.195 | 0.095 | 0.273 | -0.073 | 0.058 |
| AX | 0.959 | 0.989 | 0.961 | 0.170 | -0.234 | -0.336 |
| BX | 0.203 | 0.143 | -0.091 | 0.969 | 0.997 | 0.915 |
| ABX | 0.935 | 0.975 | 0.886 | 0.911 | 0.949 | 0.923 |

^1^Dataset: L = AX = A and X animals, BX = B and X animals, ABX = all animals (all three breeds).

^2^ Trait: LGY12 = whether a sow was inseminated for a second litter within 85 days of first farrowing, LGY15 = how many litters a sow had within 570 days of first farrowing (up to 5), Survival = repeatability trait indicating 0 if the sow survived to the next parity and 1 if she died after the current parity.

**Table S3**. Correlations between estimated breeding values for crossbred validation animals using the full dataset. Correlations are between estimated breeding values for crossbred animals when they were analyzed alone compared to using other sources of information

|  | LGY12^2^ | | | LGY15 | | | Survival | | |
| --- | --- | --- | --- | --- | --- | --- | --- | --- | --- |
| Dataset^1^ | X | AX | BX | X | AX | BX | X | AX | BX |
| A | -0.006 | - | - | 0.136 | - | - | -0.037 | - | - |
| B | 0.081 | - | - | -0.060 | - | - | 0.021 | - | - |
| AX | 0.845 | 1 | - | 0.962 | 1 | - | 0.803 | 1 | - |
| BX | 0.775 | 0.667 | 1 | 0.884 | 0.836 | 1 | 0.624 | 0.544 | 1 |
| ABX | 0.719 | 0.822 | 0.916 | 0.884 | 0.888 | 0.975 | 0.531 | 0.687 | 0.908 |

^1^Dataset: A = Breed A, B = Breed B, X = F1 crossbred of A and B, AX = A and X animals, BX = B and X animals, ABX = all animals (all three breeds).

^2^Trait: LGY12 = whether a sow was inseminated for a second litter within 85 days of first farrowing, LGY15 = how many litters a sow had within 570 days of first farrowing (up to 5), Survival = repeatability trait indicating 0 if the sow survived to the next parity and 1 if she died after the current parity.

**Table S4.** Correlations between EBVs for purebred and crossbred performance when these were considered separate

| Dataset^1^ | Trait^2^ | Breed A | Crossbreds | Breed B |
| --- | --- | --- | --- | --- |
| A+X | LGY12 | 0.73 | 0.72 | - |
|  | LGY15 | 0.96 | 0.76 | - |
| B+X | LGY12 | - | 0.45 | 0.64 |
|  | LGY15 | - | 0.90 | 0.99 |

^1^Dataset: A+X = Breed A and crossbred animals, B+X = Breed B and crossbred animals

^2^Trait: LGY12 = whether a sow was inseminated for a second litter within 85 days of first farrowing, LGY15 = how many litters a sow had within 570 days of first farrowing (up to 5)

**Table S5**. Fixed effect solutions for season on Survival in Breed B

| Season | Fixed effect estimate |
| --- | --- |
| 1 (Jan – Mar) | 0.0923 |
| 2 (Apr – Jun) | 0.0937 |
| 3 (Jul – Sep) | 0.0892 |
| 4 (Oct – Des) | 0.1033 |
